# Acute Lead Poisoning in a Cow

**Published:** 1882-07

**Authors:** George M. Parkinson


### IV.—ACUTE LEAD POISONING IN A COW.

REPORTED BY GEORGE M. PARKINSON, D.V.S.

Cow, æt. nine or ten; native stock. When first called to see the case, found a roll of lead in the stall, and inferred that she had been licking it, and possibly swallowed some of it, as it was moist and freshly fractured.

When first seen was in good flesh, and previous to this had always had a good appetite and apparently had been perfectly well.

*Symptoms.*—Severe pain, groaning, lying down, getting up, with some distention of the left lumbar region. The head hung down, and the nose was frequently pressed against the manger. Breathing sterterous, stomach full and hard. Rectum empty. Visible mucous membranes, pale in color.

*Diagnosis.*—Mild form of lead colic.

*Treatment.*—Ordered:

℞

| | |
|---|---|
| Magnesia Sulphatis, | ℥ xvi. |
| Zinc Rad. Pulv., | ℥ i. |
| Nux Vom., Fld. Ext., | Nx. xxx. |
| Mx. Aquæ, | ℈iv. |

Sig. at once.

I also gave her several enemas, and to quiet pain, chloral hydrate in ounce doses.

About three hours later her bowels commenced to move quite freely. She drank a pail of gruel, and the pain was apparently gone.

The next day, or April 13th, her temperature was 103.2° F.; pulse, sixty and irregular; respirations not disturbed. The stomach, however, remained hard, but she was up and chewing her cud. The cathartic given the night before was still acting. Appetite poor. I ordered the following:

℞

| | |
|---|---|
| Acid Sulphuric, dulci, | ℥ i. |
| Ext. Gentiane, Fld., | ℥ iv. |
| Glycerine, | ℥ iij. |

Mx. sig.; ℥ ij. in one pint of water, night and morning.

In the evening, called again and found her sufficiently improved to be discharged as convalescent.

MIDDLETOWN, CONN., April, 1882.

